# Generation of a novel model of primary human cell senescence through Tenovin-6 mediated inhibition of sirtuins

**DOI:** 10.1007/s10522-018-09792-0

**Published:** 2019-01-21

**Authors:** Hannah E. Walters, Lynne S. Cox

**Affiliations:** Department of Biochemistry, University of Oxford, South Parks Road, Oxford, OX1 3QU UK

**Keywords:** Senescence, Ageing, Sirtuin, Longevity, Tenovin-6, p21, HDAC/KDAC, SASP

## Abstract

Cell senescence, a state of cell cycle arrest and altered metabolism with enhanced pro-inflammatory secretion, underlies at least some aspects of organismal ageing. The sirtuin family of deacetylases has been implicated in preventing premature ageing; sirtuin overexpression or resveratrol-mediated activation of sirtuins increase longevity. Here we show that sirtuin inhibition by short-term, low-dose treatment with the experimental anti-cancer agent Tenovin-6 (TnV6) induces cellular senescence in primary human fibroblasts. Treated cells cease proliferation and arrest in G1 of the cell cycle, with elevated p21 levels, DNA damage foci, high mitochondrial and lysosomal load and increased senescence-associated β galactosidase activity, together with actin stress fibres and secretion of IL-6 (indicative of SASP upregulation). Consistent with a histone deacetylation role of SIRT1, we find nuclear enlargement, possibly resulting from chromatin decompaction on sirtuin inhibition. These findings highlight TnV6 as a drug that may be useful in clinical settings where acute induction of cell senescence would be beneficial, but also provide the caveat that even supposedly non-genotoxic anticancer drugs can have unexpected and efficacy-limiting impacts on non-transformed cells.

**Electronic supplementary material:**

The online version of this article (10.1007/s10522-018-09792-0) contains supplementary material, which is available to authorized users.

## Introduction

Cellular senescence is an essentially irreversible proliferation arrest mechanism induced by various stress signals, such as telomere attrition or oncogene activation, and is causally linked to ageing and the development of age-related diseases (Baker et al. 2016; Xu et al. 2018). While a multitude of signalling pathways can induce senescence, p53 is a crucial mediator, driving upregulation of p21^CDNK1A^ to induce proliferation arrest upon detection of stress (Munoz-Espin and Serrano 2014). By contrast, proteins of the evolutionarily conserved sirtuin family of histone and protein deacetylases have been reported to inhibit senescence (Hayakawa et al. 2015).

First discovered in *S. cerevisiae*, Sir2 acts as a histone deacetylase (HDAC), promoting tighter chromatin packing and reduced transcription at target loci including telomeric sequences, HM (mating type) loci and the rDNA locus RDN1 (Aparicio et al. 1991; Fritze et al. 1997). Overexpression of Sir2 has been shown to increase lifespan in yeast by 50% (Kaeberlein et al. 1999), possibly through inhibition of rDNA circle formation which may drive yeast “ageing” (Sinclair and Guarente 1997), or by mimicking the effects of caloric restriction (Chen and Guarente 2007); consistent with an anti-ageing effect of Sir2, deletion in yeast causes a lifespan reduction (Kaeberlein et al. 1999). Mice overexpressing SIRT1 in the brain have extended lifespan (Satoh et al. 2013), and small molecule SIRT1 activators SRT2104 and SRT1720 also extend lifespan (Mercken et al. 2014; Mitchell et al. 2014). The phytoalexin resveratrol is a purported activator of SIRT1 and may also enhance the binding of SIRT1 to its activator lamin A (Liu et al. 2012), a protein closely tied to lifespan regulation since a splice site LMNA mutation results in Hutchinson-Gilford progeria (Eriksson et al. 2003). However, no positive effect on longevity was detected on resveratrol feeding to mice in the highly powered multisite Interventions Testing Program (Miller et al. 2011).

Seven mammalian sirtuins have now been identified, all of which share a highly conserved NAD^+^ binding domain together with a catalytic domain, but they vary by  intracellular localization and function. Sirtuins provide a direct link between cellular metabolism and protein post-translational modifications due to their requirement for NAD^+^ for deacetylation, with the NAD^+^/NADH ratio determined by the nutritional state of the cell (Dang 2014). Of the mammalian sirtuins, SIRT1 has been the most extensively studied, acting mainly in the nucleus and deacetylating known longevity-associated targets including WRN, p53, mTORC1, NFκB, FOXO1,3,4, and PGC1α, as well as several histones (Dang 2014); several of these known targets such as p53, mTORC1 and NFκB are crucial regulators of senescence. mTORC drives geroconversion and hypertrophy in senescence (Leontieva and Blagosklonny 2016) while both mTORC and NFκB regulate the pro-inflammatory SASP of senescent cells, whereby cytokines, chemokines and matrix-remodelling enzymes are secreted with pleiotropic and pro-tumorigenic consequences (Herranz et al. 2015; Laberge et al. 2015; Rodier et al. 2009). Consistent with possible regulation by sirtuins, resveratrol treatment has been shown to suppress the SASP (Pitozzi et al. 2013). SIRT2 is mainly cytoplasmic and deacetylates some targets shared with SIRT1, as well as tubulin, PAR-3 and Cdc20 (Dang 2014).

Sirtuins are clinically important as they have been reported to play a role in retaining pluripotency of stem cells by interfering with differentiation; for example, knockdown or chemical inhibition of SIRT1 promotes neurogenesis from neural stem cells (Kim et al. 2016). In addition, sirtuin overexpression has also been linked to a number of different cancer types from leukaemia and lymphoma to solid tumours including prostate, hepatocellular and colorectal cancer (Choi et al. 2013).

Various small molecule inhibitors of sirtuins have been developed, predominantly to address the effect of sirtuin overexpression in cancer (Choi et al. 2013). Of these, tenovin-6 (TnV6), a drug initially developed in a forward chemical genetics screen to find activators of p53 (Lain et al. 2008), was shown to be an inhibitor of SIRT1/2 by haploinsuffciency profiling in yeast (Lain et al. 2008) and by fluorescence and NMR-based assays (Pirrie et al. 2012). TnV6 showed anti-tumour effects in gastric cancer cells (Hirai et al. 2014) and in CML mouse models (Yuan et al. 2012), without apparent genotoxicity. Interestingly, TnV6 is reported to induce p21 expression, through a mechanism that requires SIRT2 but is independent of p53 (McCarthy et al. 2013); this induction of p21 may account for the proliferation arrest induced by TnV6 in cancer cells (van Leeuwen et al. 2012) accompanied by only low levels of apoptosis (Sunami et al. 2013).

Since sirtuins are implicated in inhibition of senescence, we set out to determine whether SIRT1/2 inhibition by TnV6 could induce cell senescence, as a means to obtaining large numbers of cells that have entered senescence through a biologically relevant p21-dependent, non-genotoxic pathway, suitable both for biochemical analysis and potentially for screening of compounds that may modify senescent cell phenotypes. Obtaining large numbers of replicatively senescent cells is experimentally laborious, while other induction models are also problematic: oncogene induced senescence (OIS) is an acute response to high levels of *ras* overexpression, while senescence induced by DNA damaging agents intrinsically incurs a high burden of DNA damage that will impact on gene expression patterns.

Here, we report that TnV6 treatment of primary skin fibroblasts does indeed induce cellular senescence, at doses below those required to impact on proliferation of neoplastic cells. The primary cell senescence state shows elevation of p21, cell cycle arrest, increased mitochondrial load, acquisition of high levels of senescence-associated β-galactosidase, increased secretion of IL-6, indicative of SASP activation, and morphological enlargement with prominent actin stress fibres. Unexpectedly for an agent reported to be non-genotoxic, we also observed elevated DNA damage as reported by γH2AX foci.

## Results

### TnV6 suppresses HDAC activity

**Fig. 1:**
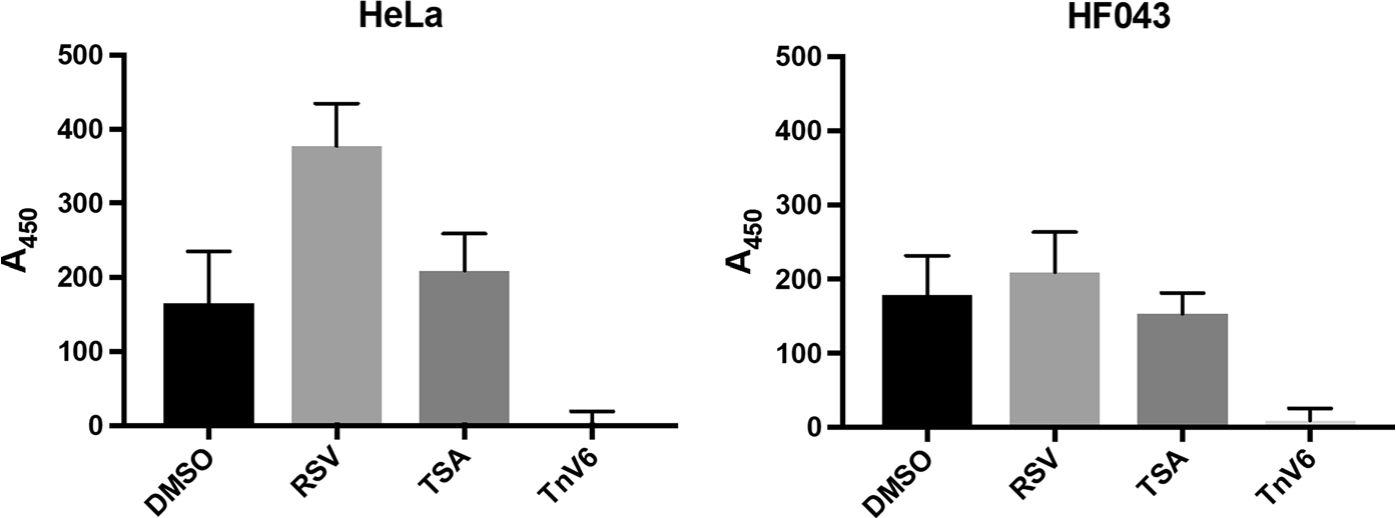
TnV6 strongly suppresses HDAC activity in both primary and cancer cells. Inhibition of deacetylase activity was measured using the Fluor de Lys^®^ HDAC fluorometric cellular activity assay (deacteylation of a substrate to generate a fluorescent product) on HeLa or HF043 cells plated in triplicate wells of 96 well plates. Cells were treated with DMSO (vehicle control), resveratrol (RSV, 50 µM), trichostatin A (TSA, 1 µM) or TnV6 (2 µM). HF043 and HeLa experiments were performed on different days (n = 2, data from one representative experiment per cell line shown; statistical analysis in Supplementary Table S1)

The drug TnV6 was originally described as an activator of p53 and developed for use as an anti-cancer agent (Lain et al. 2008); it was only subsequently found to act in a p53-independent manner as an inhibitor of SIRT1/2. To verify this activity, we employed a commercial HDAC activity assay (Fluor de Lys^®^), in which substrate deacetylation occurs within living cells, which is then assayed in cell lysates as the deacetylated substrate interacts with a developer to produce a quantifiable fluorescent signal. Proliferating primary human skin fibroblasts (HF043) and HeLa cells were incubated with TnV6 at 2 µM. Evolution of a fluorescent signal from TnV6-treated cells was compared with cells treated with resveratrol (RSV), an HDAC/SIRT1 activator, vehicle only (DMSO) negative controls and HDAC inhibitor trichostatin A (TSA), (supplied as a positive control, though notably sirtuins are insensitive to trichostatin A). Treatment with resveratrol led to increased deacetylation of the substrate in this assay, which was especially notable in HeLa cells (Fig. 1), while the positive control HDAC inhibitor TSA only led to a small decrease in deacetylation in HF043 cells at the recommended dose. However, we observed a complete ablation of deacetylation upon treatment of either HF043 or HeLa cells with 2 µM TnV6, indicative of very strong inhibition of deacetylase activity. Hence, TnV6 acts as an inhibitor of deacetylation by HDACs; given its earlier identification as a SIRT1/2 inhibitor together with our data on inhibition of deacetylation, it is likely that TnV6 acts at least in part through inhibition of SIRT1/2 in human cells.

### Low dose TnV6 treatment is cytostatic for primary cells and less toxic to cancer cells

TnV6 has been reported to halt tumour cell proliferation through inducing expression of the CDK inhibitor p21 (Jin et al. 2015). To examine whether TnV6 also blocks primary cell proliferation, primary HF043 human fibroblasts were treated with a range of concentrations of TnV6 from 100 nM to 5 µM; control cells were treated with the equivalent % of DMSO (vehicle). Cell viability was then examined by inspection of overall appearance, and by two independent assays: (i) alamarBlue (resazurin) reduction, and (ii) sulforhodamine B staining to quantify cellular biomass (Vichai and Kirtikara 2006).

**Fig. 2:**
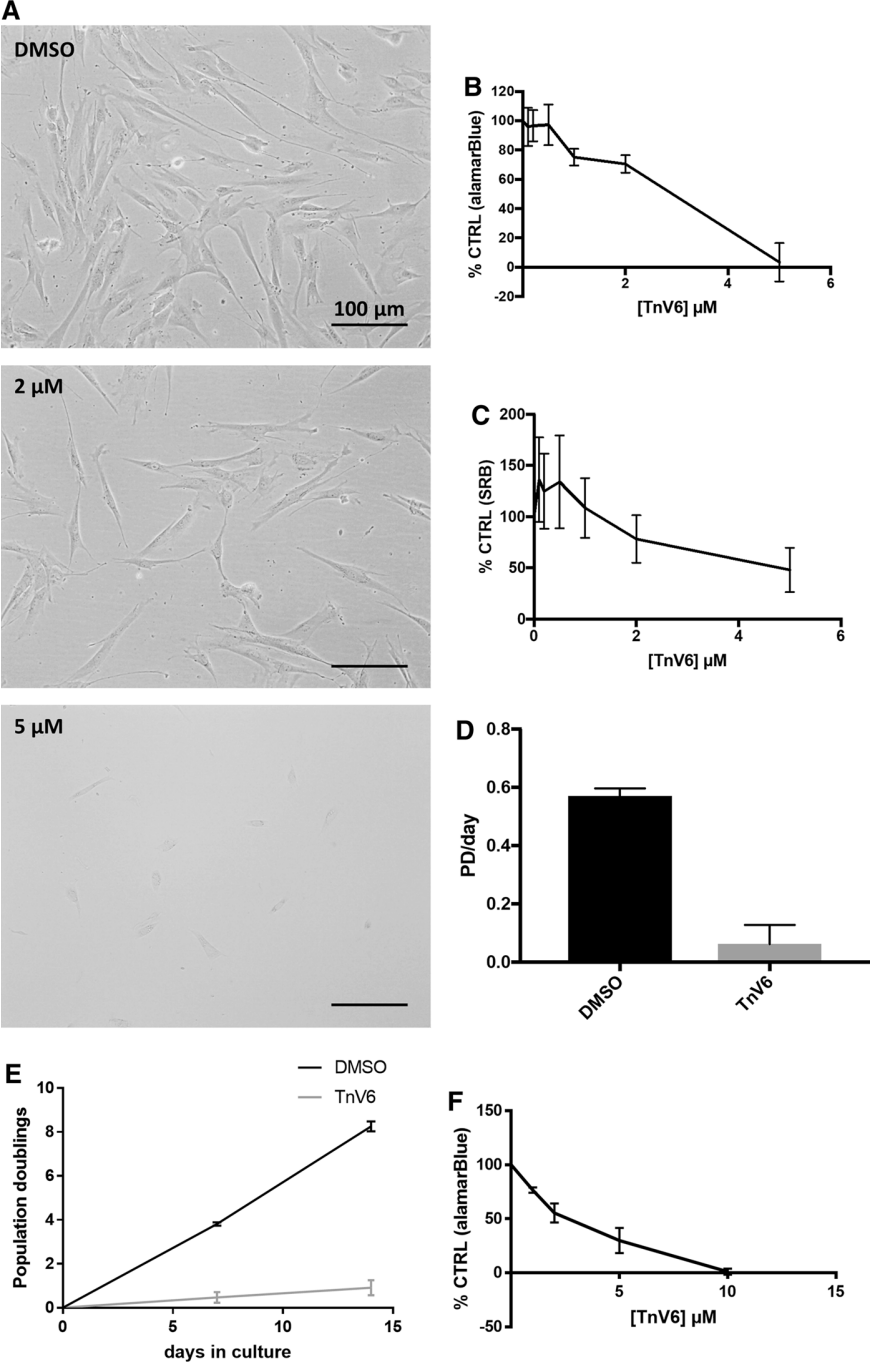
Low dose TnV6 halts proliferation in primary human fibroblasts. **a** Phase contrast microscopy images of HF043 fibroblasts (at low cumulative population doubling < 50) treated with DMSO, 2 µM or 5 µM Tnv6 for 24 h (n > 3). **b** Analysis of reducing capacity, taken as a proxy for cell viability, using the alamarBlue vital dye, following treatment of HF043 fibroblasts with TnV6 concentrations ranging from 0.1 µM to 5 µM for 72 h (n = 3, mean ± SD). **c** Total cell biomass was measured by staining with sulforhodamine B (SRB) on cells treated as in (**b**) (n = 3, mean ± SD). **d** Proliferation rate measured as population doublings (PD) per day for HF043 fibroblasts cultured with either DMSO or 2 µM TnV6 for 7 days. Cell counting was performed using a Cellometer T4 (n = 3). 2-tailed unpaired t-test *** p = 0.0002. **e** Number of population doublings over a 14-day period was measured, as in (**d**), with control cells treated with DMSO throughout, and TnV6 cells treated with 2 µM TnV6 for the first 7 days, and DMSO for days 7–14. **f** HeLa cells were treated with a range of concentrations of TnV6 for 72 h before viability analysis using alamarBlue

A strong cytotoxic effect was observed for primary HF043 skin fibroblasts within only 24 h of treatment with 5 µM TnV6; cells became detached from the substrate, rounding up and losing viability (Fig. 2a). Longer treatment for 72 h was highly toxic as shown by both alamarBlue and SRB measurement (Fig. 2b, c). Cell numbers observed microscopically after 24 h treatment with 2 µM TnV6 were decreased compared with controls (Fig. 2a), and treated cells did not exhibit the high mitotic index observed in control populations (Fig. 2a). Following 72 h treatment with 2 µM TnV6, there was a reduction both in alamarBlue and SRB signals to ~ 60% of control values (Fig. 2b, c). Combined with the microscopy findings (Fig. 2a), this may reflect proliferation arrest rather than cell death during the treatment period. This possible cytostatic effect was further assessed by treating cells continuously for 7 days with 2 µM TnV6 (or DMSO control) then directly counting cell numbers. While control populations had proliferated at a rate of ~ 0.6 populations doublings/day, the number of cells harvested after 7 days TnV6 treatment was comparable to the number of cells seeded, again suggesting a cytostatic effect of TnV6 treatment at this dose. These findings were also recapitulated in another primary human fibroblast cell line HCA2 (data not shown). To determine whether the proliferative arrest induced by TnV6 treatment was permanent, HF043 cells were again treated for 7 days with 2 µM TnV6 (or DMSO control), before directly counting cell numbers. Cells were then re-seeded without TnV6 treatment and incubated for a further 7 days, before cells were again counted to determine the rate of proliferation within the incubation window. As shown in Fig. 2e, the proliferation arrest induced by 7 day treatment with TnV6 was sustained even after treatment removal.

To determine whether cancer cells were equally sensitive to TnV6, we treated HeLa cells with a concentration range of TnV6 for 72 h before viability analysis by alamarBlue (Fig. 2f). We observed that a higher dose of TnV6 was required to elicit total cell death for HeLa cells compared with HF043 fibroblasts (10 vs. 5 µM TnV6) suggesting that primary fibroblasts are highly sensitive to TnV6 treatment compared to cancer cells.

**Fig. 3:**
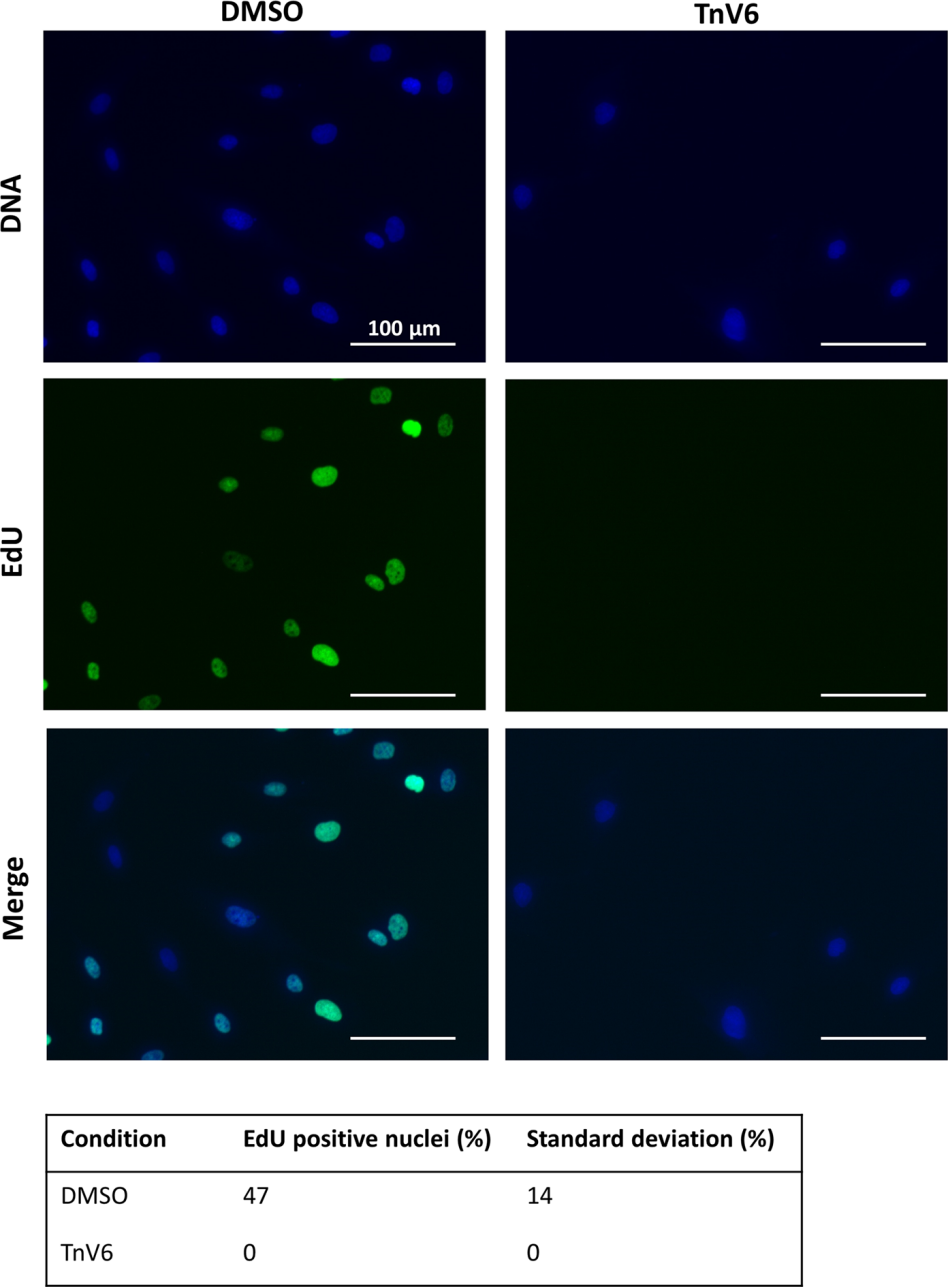
Ablation of DNA replication in primary human fibroblasts on low dose TnV6 treatment. DNA replication was assessed in HF043 fibroblasts treated for 72 h with either DMSO or 2 µM TnV6, before incubation with 10 µM EdU for 18 h, fixation and staining using Click-IT chemistry for incorporated EdU. Cells were then imaged on a ZOE^TM^ fluorescence imager with gain and contrast settings kept identical between wells and images. Fiji software was used for quantification. Technical triplicates were conducted for each experiment, with at least 50 cells analysed per well; number of biological replicates n = 3, 2-tailed unpaired t-test ** p = 0.0043

To further probe the possible cytostatic effect observed on 2 µM TnV6 treatment, we next assessed whether treatment was inhibiting cell cycle progression, using the nucleotide analogue EdU to label proliferating cells undergoing S phase. Following 72 h treatment with 2 µM TnV6 (or DMSO control), cells were then incubated for 18 h with 10 µM EdU, before fixation and staining for EdU incorporation. Nuclear EdU staining was detected in almost half of the control cells (Fig. 3, DMSO), consistent with their observed proliferation rates in routine sub-culture (Fig. 2d, PD/day ~ 0.6 for control fibroblasts). In stark contrast, none of the TnV6 treated cells showed any EdU incorporation (Fig. 3, TnV6), strongly suggesting that they had arrested in the cell division cycle without undergoing S phase (DNA synthesis).

### Primary cells arrest in G1 on TnV6 treatment

**Fig. 4:**
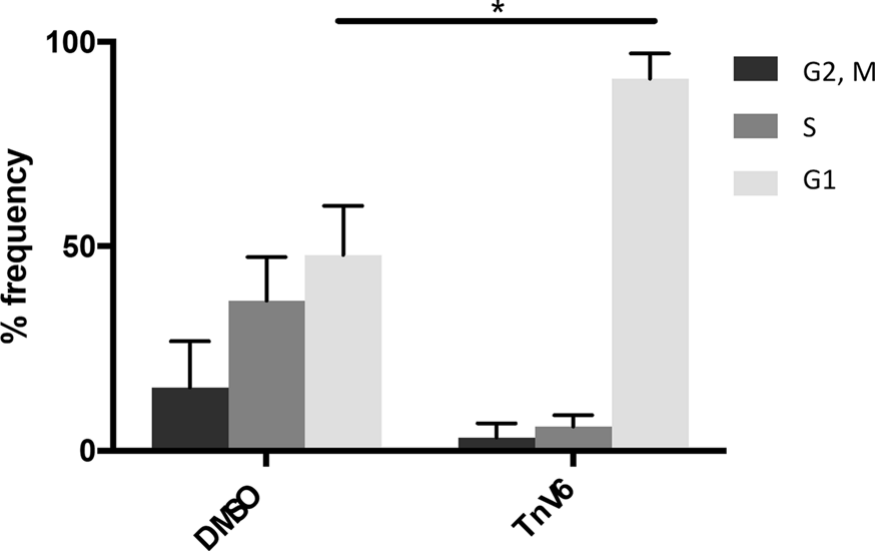
G1 phase arrest in primary human fibroblasts treated with TnV6. Cell cycle stage was assessed in HF043 fibroblasts treated with DMSO or 2 µM Tnv6 in triplicate for 72 h using the Cell Cycle Clock dye reagent. Images of stained cells were then analysed by Fiji (n = 3, > 50 cells per well analysed, data from one representative experiment shown). * p < 0.05

Our data on cell proliferation and DNA replication strongly suggested that TnV6 treatment induced cell cycle arrest. We therefore assessed cell cycle phase distribution of fibroblasts treated with TnV6 (2 µM) or DMSO as control. Primary HF043 fibroblasts were again incubated with 2 µM TnV6 (or DMSO) for 72 h before cell cycle stage was assessed using the proprietary dye Cell Cycle Clock^TM^ (Biocolour). Approximately 48% of the control cells (DMSO) were found to be in G1 phase, with 37% in S, and 15% in G2/M (Fig. 4). By contrast, very few TnV6-treated cells were in either G2/M or S phase; instead the vast majority (91%) of cells were in G1, suggesting a G1 phase cell cycle arrest upon low dose TnV6 treatment.

**Fig. 5:**
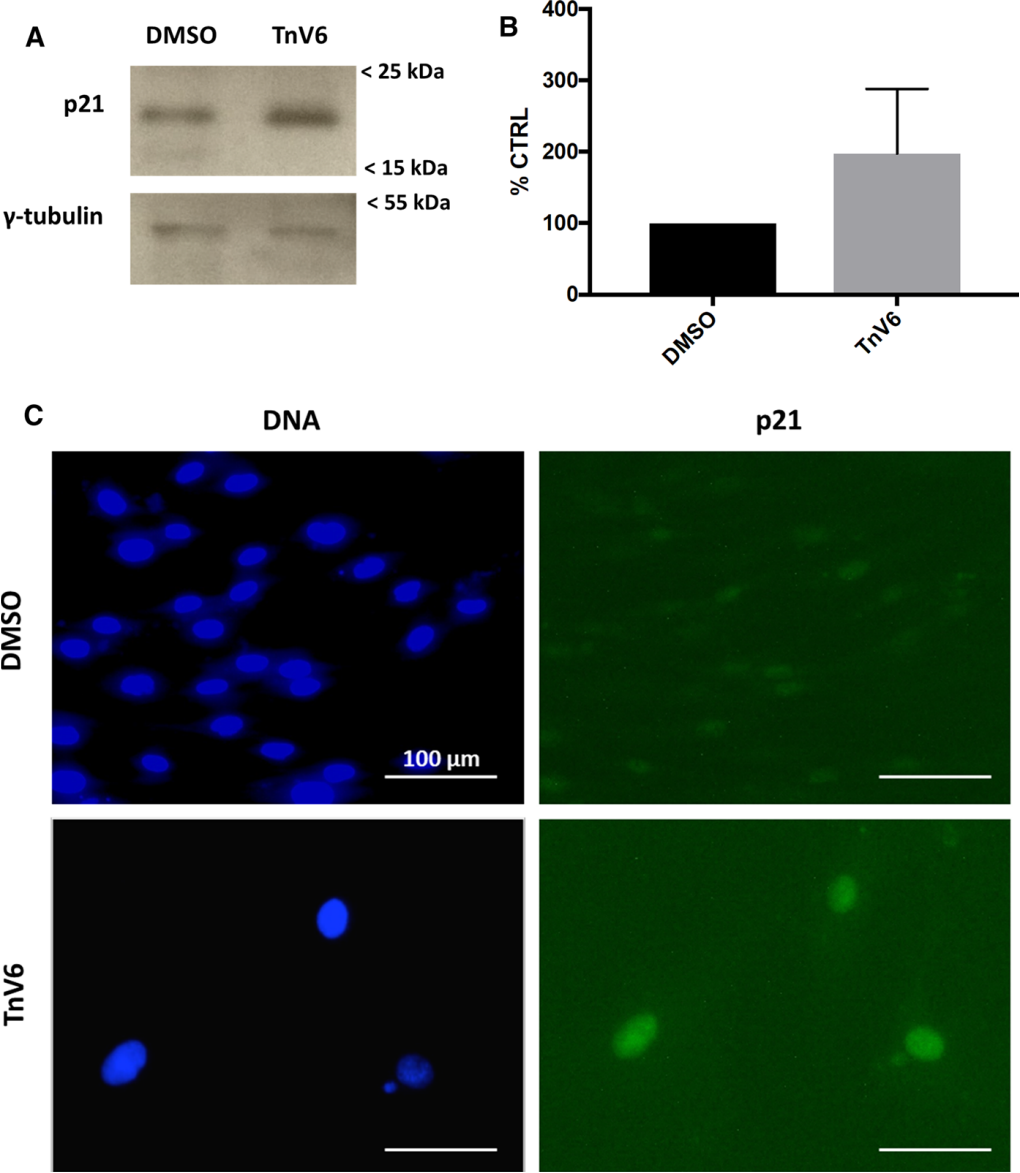
TnV6 induces p21 upregulation in primary human fibroblasts. **a** Western blot of lysates of HF043 fibroblasts treated with either DMSO (control) or 2 µM TnV6 for 7 days, probed with anti-p21 antibody (upper panel) or anti- γ-tubulin antibody (lower panel). Marker sizes shown by arrows on right. **b** Bands on the blots were quantified by densitometry using Fiji software and then the p21 signal was normalised against the tubulin loading control (n = 3). **c** Immunofluorescence of HF043 fibroblasts treated with DMSO or 2 µm TnV6 for 7 days then probed with anti-p21 antibody and Alexafluor-488 secondary antibody, with DNA counterstained with NucBlue live

To determine the basis of this G1 arrest, we then probed treated cells for the presence of the cyclin kinase inhibitor p21. Cell lysates were probed by Western blotting for p21 (Fig. 5a), and levels quantified by densitometry, with normalisation against the loading control tubulin (Fig. 5b). A marked increase in p21 was detected following 3-day exposure of cells to TnV6. Similarly, immunofluorescence of HF043 fibroblasts treated for 7 days (or DMSO controls) showed strong nuclear staining for p21 after TnV6 treatment that was not detected in the vehicle-only controls (Fig. 5c). Interestingly, we noted the presence of extra-nuclear DAPI-staining in a subset of TnV6-treated cells suggestive of cytoplasmic chromatin fragments found in senescent cells.

### Extended TnV6 treatment leads to increased cell size and senescent morphology in primary fibroblasts

Since elevated p21 together with lack of proliferation are features of both transiently arrested and senescent cells, we next assessed whether extended exposure to TnV6 would lead to morphological changes associated with cell senescence. We treated cells continuously for 7 days with 2 µM TnV6 prior to analysis for phenotypes of senescence, since morphological changes on induction of senescence by experimental DNA damage or oncogene activation only begin to manifest about 1 week after induction.

**Fig. 6:**
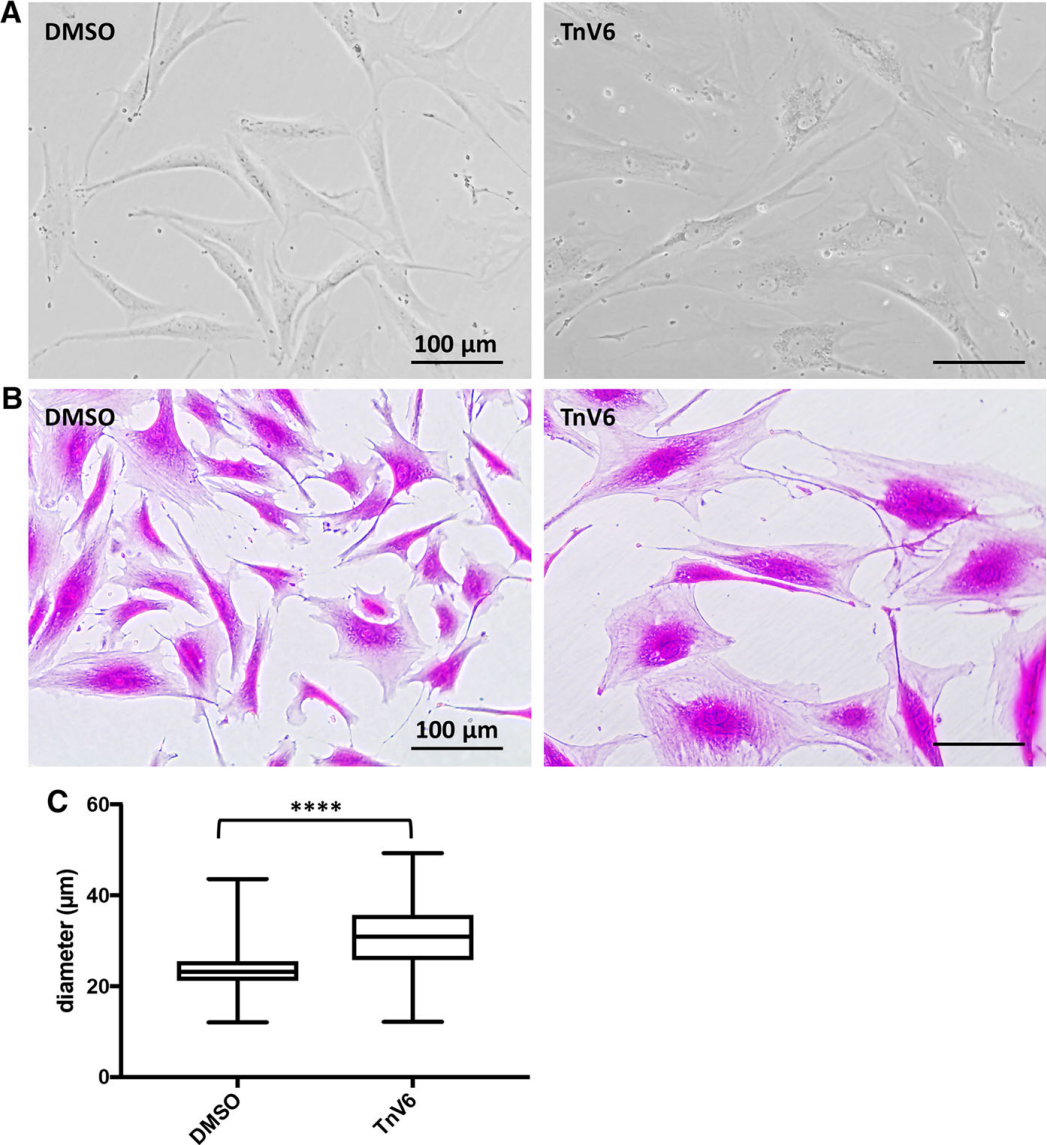
Extended TnV6 treatment induces senescent morphology. **a** Phase contrast microscopy of HF043 fibroblasts treated with DMSO or 2 µM TnV6 for 7 days (n > 3). **b** Cells as in **a** were fixed and stained with sulforhodamine B then imaged using transmission light microscopy (n > 3). **c** Cell diameter was analysed for cells treated as above, with > 30 cells counted per sample (n > 3 biological replicates, results from one representative experiment shown). Box shows 25th and 75th percentiles with median line; bars show maximum and minimum, **** p < 0.001

Phase contrast microscopy revealed that control cells (DMSO only) maintained classic fibroblast spindle morphology throughout the experiments, whereas TnV6-treated cells became dramatically enlarged and showed granular inclusions (Fig. 6a); these morphological differences were especially apparent after staining with SRB, with TnV6-treated cells also appearing flattened (Fig. 6b).

To quantify this increase in cell size, we harvested the cells and measured their diameter in suspension, in order to overcome possible issues of flattening and spreading which indicate area only and may give an over-estimate of overall cell size/volume. We observed a statistically significant increase in cell diameter in suspension following TnV6 treatment. Further, consistent with the increased heterogeneity associated with biological ageing and senescence, there was greater variation in the cell size distribution compared with control cells (Fig. 6c).

### Markers of cell senescence are elevated on TnV6 treatment

**Fig. 7:**
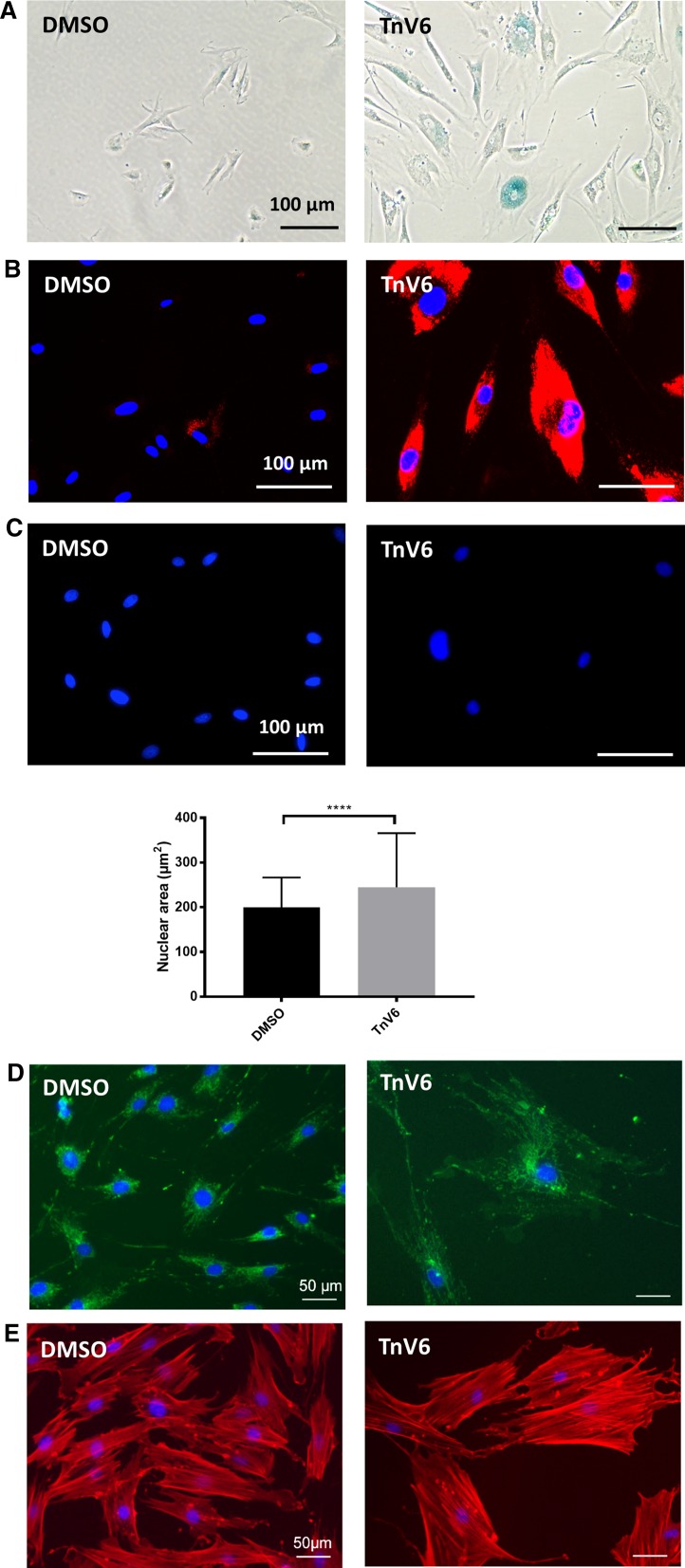
Senescence phenotypes result from long term TnV6 exposure. **a** SAβGAL staining of HF043 fibroblasts treated for 7 days with DMSO or 2 µM TnV6. **b** Lysosomal analysis by Lysotracker Red staining of HF043 fibroblasts treated for 72 h with DMSO or 2 µM TnV6; DNA was counterstained with NucBlue Live. Images were acquired in living cells on a ZOE fluorescence imager with gain and contrast settings constant throughout. **c** Nuclear area was analysed from fluorescence microscopy images of cells stained with NucBlue Live and quantified by Fiji (n > 3, > 50 nuclei counted per sample, data from one representative experiment shown). **** p < 0.001. **d** Mitochondrial staining of HF043 fibroblasts after 7-day treatment with DMSO or 2 µM TnV6 using Mitotracker Green and imaged live (n = 2). **e** Rhodamine-phalloidin staining for actin in HF043 fibroblasts fixed and stained after 7-day treatment with DMSO or 2 µM TnV6. DNA was counterstained with NucBlue Live (n = 2)

The increase in cell size and granularity, together with cell cycle arrest and p21 elevation were suggestive that TnV6 treatment might be driving cells into senescence. We next examined the lysosomal content of treated and untreated cells using Lysotracker Red (a pH-dependent lysosomal fluorescent stain) alongside the canonical marker of senescence, lysosomal β-galactosidase activity at pH 6.0 (Dimri et al. 1995). We found that treated cells had dramatic increases in both SAβGAL (Fig. 7a) and Lysotracker staining (Fig. 7b), compared to the controls (DMSO). These changes are indicative of lysosomal stress and induction of senescence.

Notably, we also observed a statistically significant increase in mean nuclear area from 200 µm^2^ in control cells to 245 µm^2^ which occurred after only 72 h TnV6 treatment (Fig. 7c). This finding is consistent with chromatin decompaction, a likely consequence of TnV6 inhibition of sirtuin-mediated histone deacetylation. Since this nuclear enlargement occurs within just 72 h of treatment i.e. before the onset of other senescence-associated morphological changes, it is possible that a changing chromatin landscape resulting from persistent histone acetylation may drive alterations in gene expression that result in senescence-specific changes.

Cellular mitochondrial load is known to increase in senescence, either as a compensatory mechanism for increasingly inefficient mitochondrial activity, or playing a causative role in driving senescence (Correia-Melo et al. 2016). We therefore assessed mitochondrial load in treated and untreated living cells using Mitotracker Green. We observed a dramatically increased and highly reticular mitochondrial load following TnV6 treatment, compared to control proliferating fibroblasts (DMSO alone) which exhibited a low mitochondrial load, with the majority of cells showing a perinuclear distribution of mitochondria (Fig. 7d).

A further biomarker of senescence is the presence of filamentous actin stress fibres. We therefore used rhodamine-phalloidin to visualize cellular actin after prolonged exposure to TnV6. A marked increase in senescent-like actin stress fibres was observed in cells treated with TnV6 compared with vehicle-only control cells, which showed more diffuse actin patterns characteristic of proliferating cells (Fig. 7e).

### Extended TnV6 treatment induces SASP secretion by primary fibroblasts

Senescent cells secrete a broad profile of pro-inflammatory cytokines, chemokines, matrix-remodelling enzymes and growth factors (the SASP), which has pleiotropic signalling consequences; as well as alerting the immune system to the presence of senescent cells for clearance, the SASP can induce senescence in neighbouring cells, and have pro-tumorigenic signalling effects including promoting EMT (Laberge et al. 2012). The SASP may therefore underlie many of the deleterious effects of senescent cell accumulation in vivo (Baker et al. 2016; Xu et al. 2018). To test whether extended TnV6 treatment could induce SASP secretion, we chose to use IL-6 as a marker for the SASP, both because IL-6 is known to be a consistent and robustly secreted component of a highly heterogeneous SASP, and because IL-6 itself is implicated in pro-tumorigenic SASP signalling (Ortiz-Montero et al. 2017).

**Fig. 8:**
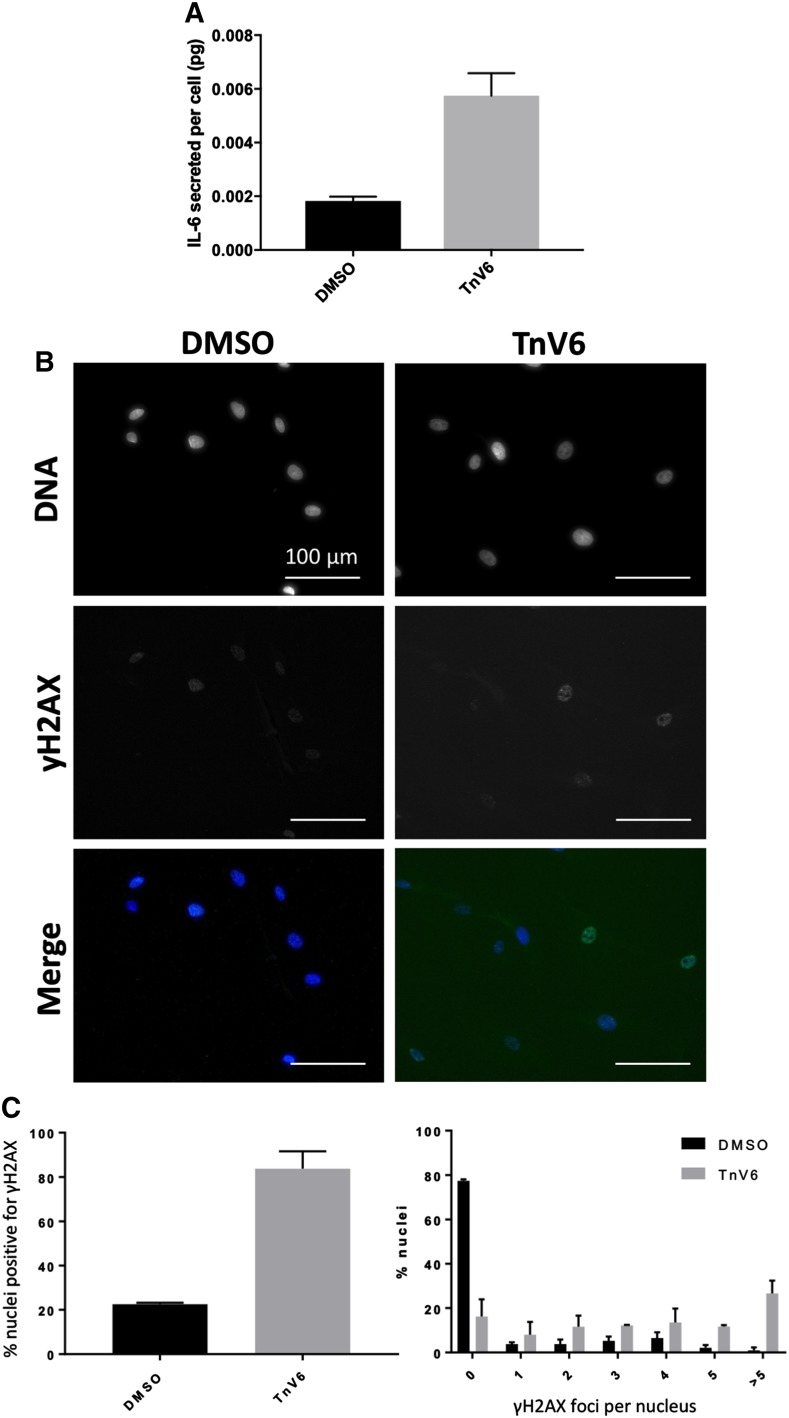
IL-6 is upregulated in TnV6-treated cells and associates with elevation of DNA damage. **a** ELISA was used to measure IL-6 secreted into 24-hour conditioned medium by HF043 fibroblasts treated with 2 µM TnV6 or DMSO for 7 days in total. Recombinant human IL-6 was used to generate a standard curve for determination of IL-6 amounts, and IL-6/cell (pg) was calculated following cell counting. n = 3, mean ± S.D. are shown, 2-tailed unpaired t-test p = 0.0059. **b** Analysis of DNA damage by immunofluorescence for γH2AX in HF043 fibroblasts treated for 7 days with 2 µM TnV6 or DMSO, with DNA counterstained by NucBlue Live. **c** Quantitation of  % nuclei with γH2AX staining (2-tailed unpaired t-test p = 0.0002) and number of γH2AX foci per nucleus. Mean ± S.D. shown

To measure IL-6 secretion from TnV6-treated and control fibroblasts, levels of IL-6 in 24 h conditioned media taken from treated and untreated cells were measured by ELISA. Results were quantified using a standard curve generated from purified recombinant human IL-6 protein, and absolute values in terms of IL-6 secreted per cell were calculated by dividing interpolated IL-6 concentrations by total cell numbers, which were determined by performing direct cell counting after harvesting of conditioned medium (Fig. 8a). While very little IL-6 was detectable in the medium of proliferating fibroblasts (DMSO controls), we observed a 3.5 fold elevation in IL-6 secreted by cells treated for 1 week with low dose (2 µM) TnV6, indicative of upregulation of the SASP on TnV6 treatment.

### Unexpected DNA damage following TnV6 treatment of primary cells

TnV6 has been reported to have no genotoxic effects in transformed cancer cell lines (MacCallum et al. 2013). However, our data above show that it causes senescence in primary fibroblasts including secretion of IL-6, a canonical marker of the SASP, together with markers such as increased mitochondrial load. While no mechanism is known for TnV6 to induce DNA damage directly, autocrine feedback loops that reinforce senescence are activated by SASP signalling upon senescence induction that can induce DNA damage through release of mitochondrial ROS (Passos et al. 2010), and senescent cells display a chronic DNA damage response (Fumagalli et al. 2014), which can both initiate and maintain the growth arrest. Alternatively, chromatin decompaction may lead to accumulation of DNA breaks, as shown for the HDAC inhibitor TSA in Werner syndrome cells (Turaga et al. 2007). We therefore examined levels of the DNA damage marker γH2AX by immunofluorescence in HF043 fibroblasts after 7 days exposure to TnV6. While only 20% of control cells showed γH2AX positive staining, which was of low intensity and not localised to specific subnuclear foci, the vast majority (~ 85%) of TnV6-treated cells were positive for γH2AX, with multiple foci per nucleus (Fig. 8b and quantitation in Fig. 8c). Hence treatment with TnV6 for as little as 7 days results in a persistent DNA damage signal despite previous data suggesting TnV6 is not directly genotoxic.

## Discussion

In this paper, we have demonstrated that pharmacological inactivation of SIRT1/2 by low dose treatment with the drug Tenovin-6 results in onset of cellular senescence in primary human skin cells. The role of sirtuins in ageing has for some years been controversial. While Sir2 overexpression increases yeast lifespan and mutation decreases lifespan (Kaeberlein et al. 1999), and studies in worms and flies suggest that sirtuins act as pro-longevity factors (Wood et al. 2004), it has also been argued that such studies are dependent on factors other than the sirtuins (Burnett et al. 2011). For example, metabolic activators and/or downstream components of sirtuin activity (e.g. nicotinamide mononucleotide) have been reported to improve muscle strength and improve certain ageing phenotypes in mice (Imai and Guarente 2016). However, the mechanistic basis of the impact of a deacetylase on ageing has not been clearly elucidated. Here, we provide strong evidence that sirtuins are active in protecting against ageing by delaying the onset of cellular senescence, and that their inhibition promotes premature cellular senescence.

Our results underline a link between sirtuin signalling and senescence, and although our findings are consistent with previous data showing TnV6 upregulation of p21 (Lain et al. 2008), exactly how the sirtuins regulate cell proliferation is not yet clear. Both SIRT1 and SIRT2 are pleiotropic in terms of cellular targets: SIRT1 deacetylates nuclear proteins including p53, mTORC1, PI3K, FOXO1,3,4, PCG1α, HSF1 and NFκB as well as several histones, while SIRT2 also targets some of these proteins in the cytoplasm, as well as tubulin, PAR-3 and Cdc20 (Dang 2014). A large proportion of these targets have well-documented roles in senescence, for example mTOR is a biochemical nexus for senescence signalling, inhibition of which reverses phenotypes of senescence (Leontieva and Blagosklonny 2016; Walters et al. 2016). Moreover, mTORC1 and NFκB both regulate the SASP (Herranz et al. 2015; Laberge et al. 2015); this signalling axis may be responsible for the upregulation of IL-6 that we observe following prolonged TnV6 treatment. Further, p53 is a critical regulator of proliferation, senescence and apoptosis, and its interaction with another SIRT1 target (FOXO4) may regulate entry into senescence (Baar et al. 2017). Over-active p53 promotes ageing (Rodier et al. 2007), while deacetylation, possibly by sirtuins, may damp down p53’s pro-ageing activity; TnV6 may block this deacetylation to promote senescence onset. Indeed, we demonstrate in a fluorimetric assay that TnV6 treatment of cells leads to reduced deacetylation of an assay substrate. However, we cannot exclude the possibility that TnV6 may also inhibit the activity of non-sirtuin deacetylases. This could be further explored experimentally by investigating any antagonism of NAD^+^ supplementation on TnV6-mediated sirtuin inhibition, as SIRT1 and SIRT2 are NAD^+^-dependent deacetylases. It will also be important to investigate the relative contributions of SIRT1 and 2 in preventing premature senescence, through genetic manipulation or direct targeting using further inhibitors such as the SIRT2-specific Tenovin-D3.

The observation of γH2AX foci, and by inference, sites of DNA damage and a persistent DNA damage response (DDR) in TnV6-treated cells is intriguing. It is well documented that senescent cells exhibit a chronic DDR (Fumagalli et al. 2014) responsible for establishing and maintaining senescence. Furthermore, retrograde signalling by TGFβ as a component of the SASP increases cellular ROS, causing a DDR that deepens the state of senescence (Passos et al. 2010). We observed increased IL-6 secretion upon extended treatment with TnV6 and an increased mitochondrial load, suggestive of an active SASP and possibly of increased ROS. This could constitute a possible mechanism for the observed DNA damage caused by a supposedly non-genotoxic agent (Lain et al. 2008). However, SIRT1 may also have a role in DNA repair, promoting homologous recombination (Palacios et al. 2010), meaning that SIRT1 inhibition could compromise cellular DNA repair capacity. Interestingly, SIRT1-mediated homologous recombination may require WRN, a helicase and exonuclease mutated in the premature ageing Werner syndrome. Replication stress resulting from WRN mutation (Pichierri et al. 2001; Rodriguez-Lopez et al. 2002) may itself drive cellular senescence by triggering a DNA damage response and highly premature senescence is characteristic of Werner syndrome patient-derived cells. Interestingly, a link between upregulation of WRN and SIRT1 was observed in calorically restricted rats, with in vitro investigation suggesting that sirtuin-mediated deacetylation may stabilize WRN levels (Kahyo et al. 2008).

Cellular senescence is also accompanied by marked changes in chromatin, mediated by epigenetic changes including CpG methylation and histone modifications. Acting as histone deacetylases, the sirtuins broadly promote closer chromatin packing. We observed a significant increase in nuclear area increase after only 72 h treatment with TnV6, consistent with the idea that blockade of sirtuin-mediated histone deacetylation allows chromatin decompaction, which is likely to be accompanied by changes in gene expression patterns. Of note, other HDAC inhibitors may induce p21-driven senescence, including TSA (Okamoto et al. 2006). Significant alterations in nuclear size, lamina structure (Lenain et al. 2017), and chromosome localization (Bridger et al. 2000) occur in senescence. As we observed that TnV6 induces a G1 phase arrest, this increase in nuclear area is not simply due to a higher proportion of nuclei with a 4n (replicated) DNA complement. Instead, this increase in nuclear area is likely to be due to increased histone acetylation and relaxed chromatin packing.

Recent landmark studies have confirmed that the presence and accumulation of senescent cells during ageing drives ageing and age-related diseases: selective depletion of senescent cells in a naturally aged mouse caused dramatic rejuvenation (Baker et al. 2016), while introduction of senescent cells into young mice drives ageing pathologies (Xu et al. 2018). The field of senescence research is exploring different avenues in which modulating or killing senescent cells in vivo could be therapeutically beneficial, for example, during ageing or after genotoxic therapies. In vitro work on senescence uses a range of different models of senescence, including oncogene-induced senescence (OIS), replicative senescence (RS) and DNA damage-induced senescence, hence a variety of different biochemical pathways are being experimentally manipulated to induce senescence. However, the resulting senescent states are not interchangeable: recent reports show major differences in chromatin profiles, transcriptomes and proteomes between models. Further, it is not clear which model, or which combination of models, is the most relevant for the study of human ageing in vivo. We propose TnV6 as a further experimentally amenable tool to investigate the biochemistry of cellular senescence, through manipulation of sirtuins which are already implicated in senescence and ageing.

TnV6 has been developed as a promising anti-cancer agent, toxic at micromolar concentrations in tumour cell lines (Hirai et al. 2014; Jin et al. 2015). However, we observed toxicity in primary cells at doses to which cancer cells were less sensitive. Even low doses, which did not appreciably impact on cancer cells, were cytostatic for primary cells. Crucially, the concentrations we found to be toxic or cytostatic in our human fibroblast model are lower than those shown to be effective in tumour cells (Hirai et al. 2014), and HeLa cells in our hands. Furthermore, our novel finding that TnV6 induces cellular senescence in primary human fibroblasts has significant implications for clinical use of this drug in cancer chemotherapy—inducing senescence in healthy neighbouring cells could drive premature ageing, secondary cancer formation and promote metastasis through the SASP. Indeed, the drug zidovudine used in HAART treatment for HIV patients, which has been shown to induce senescence in primary cells, may have caused premature ageing in patients through induction of senescence (Torres and Lewis 2014). It is therefore necessary to be cautious with novel cancer therapies that may impinge on biochemical pathways implicated in cell and organismal ageing.

However, although senescence has become synonymous with pathological ageing, it is important to remember that it also has beneficial physiological roles in development, wound healing and regeneration, as well as providing a barrier to tumorigenesis. Therefore, in certain contexts, agents that induce senescence may be therapeutically useful. For example, and crucially only if delivery could be targeted to avoid healthy neighbouring cells, TnV6 could be used to induce senescence in tumour cells, or to induce senescence and promote regeneration in liver, cardiac and renal fibrosis, in skin wound healing or to prevent development of atherosclerotic plaques (Munoz-Espin and Serrano 2014). Hence, we propose that Tenovin-6 may retain some clinical utility by exploiting its ability to induce senescence.

## Methods

### Cell culture

HF043 diploid neonatal primary human fibroblasts were obtained from Dundee CELL products, while HeLa cells were a gift from Prof. F. Barr (University of Oxford). Cells were routinely subcultured in DMEM (Sigma) with 10% FCS (Gibco) and incubated in a humidified incubator at 37 °C with 5% CO_2_. Cells were imaged using an EVOS digital microscope (Life Technologies) and routinely sub-cultured according to standard protocols (Walters et al. 2016). For diameter analysis and calculation of proliferation rates by cell counting, 20 µl of cell suspension was taken during cell harvesting for analysis using a Cellometer T4 (Nexelcom). The primary cells used in all experiments were at low CPD (cumulative population doubling, < 50 CPD c.f. HF043 fibroblasts reach replicative senescence around CPD 90 (Walters et al. 2016)).

### Drug treatment

Tenvoin-6 (Dundee CELL products) was stored as a 10 mM stock in DMSO at − 20 °C. Prior to drug treatment, cells were seeded into filter-capped T25 flasks or CELLstar plates (Greiner), allowed to bed down overnight, then medium was removed, cells washed in PBS, and fresh medium supplemented with drug added at stated concentrations (maximum final DMSO concentration 0.1%).

### Western blotting

Cells grown in T25 flasks were harvested by gentle trypsinization, pelleted by centrifugation (6 kRPM, 1 min), then washed in 1 ml PBS and re-centrifuged and buffer aspirated. The resulting pellet was resuspended in 30 µl RIPA buffer (Thermo Scientific) supplemented with 1:100 protease/phosphatase inhibitor cocktail (Cell Signalling). Lysates containing equal numbers of cells were then heated for 5 min at 95 °C in 1X NuPAGE LDS buffer (Novagen) containing 100 mM DTT and benzonase nuclease (1:200 v/v) then proteins were separated on 10% Bis–Tris NuPage gels (Invitrogen) at 150 V for 1–1.5 h with MOPS-SDS running buffer (Invitrogen). Transfer to nitrocellulose was performed using a BioRad Trans-Blot Turbo system (7 min, 1.3 A, 17 V). Blots were blocked in 5% BSA in PBS-T for ≥ 1 h, washed twice in PBS, then probed with primary antibody in PBS-T-BSA for ≥ 1 h (RT). Following five washes (2 min per wash) in PBS-T, blots were probed with HRP-conjugated secondary antibodies in PBS-T-BSA for ≥ 1 h. Subsequently, blots were washed four times in PBS-T then once in PBS. The EZ-ECL kit (Biological Industries) was used according to manufacturer’s instructions with Hyperfilm MP X-ray film (Amersham) and Xograph compact X4 automatic processor (Xograph, UK). Antibodies used were α-p21 Ab109199 (Abcam), α-γ-tubulin T5192 (Sigma-Aldrich) and HRP-conjugated α-rabbit IgG P0448 (Dako), all at 1:1000.

### Fluor de Lys^®^ HDAC assay

The fluor-de-lys HDAC fluorimetric cellular activity assay was performed according to manufacturer’s instructions (Enzo) with luminescence readings taken using a Pherastar FS platereader (BMG).

### AlamarBlue viability assay

The alamarBlue assay was performed according to manufacturer’s instructions (Thermo Scientific), adding the 10x  stock at 1:10 dilution to obtain 1x final concentration in DMEM-FCS and assessing fluorescence after 4 h incubation (Ex570, Em585 using a BMG Pherastar FS platereader).

### Sulforhodamine B (SRB) biomass and morphology assay

The SRB biomass assay was performed according to (Vichai and Kirtikara 2006) with addition of 10 mM Tris for dye solubilisation; quantification was carried out using a Pherastar FS platereader reading absorbance at 510 nm (BMG). For microscopy, cells were imaged prior to solubilisation of the dye.

### Determination of cell cycle stage

Cells were harvested from 24-well plates after drug treatment by trypsinization with TrypleExpress, with quenching with DMEM-FCS. Cells were then pelleted in a microfuge for 30 s at 6 kRPM, before resuspension of the pellet in 1:3 Cell Cycle Clock^TM^ Reagent (Biocolor) in DMEM-FCS, and incubation for 15 min (RT). The suspension was then re-pelleted, resuspended in 250 µl DMEM-FCS, placed into wells of a 12 well plate then imaged immediately. The photomicrographs were analysed by Fiji, by thresholding according to manufacturer’s instructions. Assays were only conducted on sub-confluent cells to avoid confounding effects of contact inhibition inducing quiescence.

### SAβGAL staining

SAβGAL staining was performed using the Senescence β-Galactosidase Staining Kit #9860 according to manufacturers’ instructions (Cell Signalling).

### DNA replication analysis

Click it EdU labelling and detection was performed according to manufacturer’s instructions (Invitrogen) with continuous label (18 h) EdU incorporation (10 µM).

### Lysosome and mitochondria staining

Cells were incubated with DMEM-FCS containing either 50 nM Lysotracker Red or 1 nM Mitotracker Green according to manufacturer’s instructions (Life Technologies, both for 30 min at 37 °C); media was replaced with fresh DMEM-FCS prior to fluorescence microscopy.

### Actin staining

For actin visualisation, cells were fixed in 3.7% formaldehyde (10 min, RT), permeabilised in 0.1% Triton X-100 in PBS (10 min, RT) and then stained with rhodamine phalloidin (6.6 nM) for 40 min, RT, in the dark. Cells were washed and imaged in fresh PBS.

### Immunofluorescence

Cells were fixed in 3.7% formaldehyde (10 min, RT), then blocked in 5% donkey serum (Dako) in PBS. Primary antibody (1:200 in PBS with 0.3% Triton X-100 and 1% BSA) was incubated overnight (4 °C, humidified chamber), cells washed twice in PBS and then incubated with secondary antibody overnight (1:1000 in PBS with 0.3% Triton X-100 and 1% BSA). Cells were washed twice in PBS before imaging. Antibodies used were α-γH2AX 9718 (CST), α-p21 Ab7960 (Abcam) and Alexafluor-568 α-rabbit IgG A11011 and Alexafluor-488 α-mouse IgG A-11011 (both Invitrogen).

### Imaging

An EVOS digital microscope and BioRad ZOE fluorescence imager were used for all microscopy. Where appropriate, DNA was counterstained with NucBlue Live Ready Probes Reagent (Life Technologies) according to manufacturer’s instructions.

### Statistical analysis

Statistical analysis and figure production was performed using GraphPad Prism software, with unpaired student t-tests used to analyse nuclear area and cell diameter data. Error bars on all graphs are standard deviation.

## Electronic supplementary material

Below is the link to the electronic supplementary material.

Supplementary material 1 (DOCX 47 kb)
