# Supplementary material for: Generation of a novel model of primary human cell senescence through Tenovin-6 mediated inhibition of sirtuins
Source: Biogerontology. 2019 Jan 21;20(3):303–19. doi: 10.1007/s10522-018-09792-0 (PMC6535423; doi:10.1007/s10522-018-09792-0)
Supplement: Supplementary file 1 — Supplementary material 1 (DOCX 47 kb) [file 10522_2018_9792_MOESM1_ESM.docx]

# HeLa

| HeLa | DMSO | RSV | TSA | TnV6 |
| --- | --- | --- | --- | --- |
| DMSO |  | 0.0049 | ns | 0.0199 |
| RSV | 0.0049 |  | 0.0183 | <0.0001 |
| TSA | ns | 0.0183 |  | 0.0052 |
| TnV6 | 0.0199 | <0.0001 | 0.0052 |  |

# HF043

| HF043 | DMSO | RSV | TSA | TnV6 |
| --- | --- | --- | --- | --- |
| DMSO |  | ns | ns | 0.0040 |
| RSV | ns |  | ns | 0.0014 |
| TSA | ns | ns |  | 0.0105 |
| TnV6 | 0.0040 | 0.0014 | 0.0105 |  |

# Supplementary Table 1: Statistical analysis of Fluor de Lys® assay in Figure 1. P values given for ANOVA with Tukey test for multiple comparisons (all means compared).
